# Performance and Mechanism of Hydrolyzed Keratin for Hair Photoaging Prevention

**DOI:** 10.3390/molecules30051182

**Published:** 2025-03-06

**Authors:** Jiayi Fan, Lei Wu, Jing Wang, Xiaoying Bian, Chongchong Chen, Kuan Chang

**Affiliations:** 1 Key Laboratory of Synthetic and Biological Colloids, Ministry of Education, School of Chemical & Material Engineering, Jiangnan University, Wuxi 214122, China; 6220610019@stu.jiangnan.edu.cn (J.F.); 6240610040@stu.jiangnan.edu.cn (L.W.); jingwang@jiangnan.edu.cn (J.W.); 2 Jiangnan Institute of Beauty Research, Wuxi 214122, China; 3 Unilever (China) Investing Co., Ltd., Shanghai 200335, China; marina.bian@unilever.com (X.B.); hilary.chen@unilever.com (C.C.)

**Keywords:** hair photoaging, UV damage, hydrolyzed keratin

## Abstract

Photoaging is common and represents one of the primary pathways for hair damage in daily life. Hydrolyzed keratin, which is usually derived from wool and consists of a series of polypeptide molecules, has been investigated as a UV damage prevention ingredient for hair care. Scanning Electron Microscopy (SEM) and fluorescent penetration experiments verified that hydrolyzed keratin can deposit on the hair cuticles to form a film and partly penetrate into the hair cortex. This film played as a UV reducer and helped hair resist surface damage and maintain a sleek and healthy morphology after UV radiation. Surprisingly, it was found that hydrolyzed keratin treatment combined with subsequent UV radiation could significantly improve the tensile properties of hair. For hydrolyzed-keratin-treated hair, tensile strength was maintained after UV radiation, while, as a comparison, it decreased by 14.32% for untreated hair. This phenomenon is explained by a UV-induced degradation–penetration mechanism. During UV radiation, an increase in free amino acid content and conductivity was observed for the hydrolyzed keratin solution, demonstrating photodegradation into smaller peptides and amino acids. The degradation of hydrolyzed keratin allowed it to more easily enter the interior of the hair cortex, thereby enhancing its tensile properties by enhancing the chemical bonds.

## 1. Introduction

Hair damage is a common phenomenon caused by various factors which has been attracting more and more research interest in recent years. Various factors in the processes of perming, straightening, and dyeing, including oxidizing agents [1,2,3], reducing agents [4], and heat [5], can cause certain damage to the hair cuticle and cortex and lead to irreversible changes on the hair’s surface and internal structure. In addition to these human-induced damages, hair damage also occurs naturally every day due to a diverse range of environmental disruptors like ash, air pollutes, and, more importantly, UV radiation. Among these environmental disruptors, UV radiation from sunlight has a rather severe impact on the physical and chemical properties of hair. Considering that UV radiation is of significance to human health, which plays an important role in the activation of local neuro-immuno-endocrine sensing and regulatory mechanisms [6,7], it is, therefore, more important to explore methods to prevent photodamage rather than fully avoid exposure to UV radiation.

Hair damage induced by UV radiation is also called hair photoaging, which could lead to many types of hair damage such as protein loss [8], amino acid and lipid oxidation [9,10], melanin degradation [11], and obvious cuticle deformation [12]. For instance, Richena et al. employed Atomic Force Microscopy (AFM) to characterize hair surface after UV irradiation and found that the average step height of the surface cuticle layer of the hair significantly increased from 203 nm to 338 nm after irradiation [12]. In addition to surface damage, the mechanical properties and manageability of hair are also greatly influenced under UV radiation, as demonstrated in a study by Nogueira et al. It was found that, after photoaging, both the tensile strength and largest elongation of hair showed a noticeable decrease [1]. Photoaging would be more severe for dyed hair due to more cysteic acid created during hair coloring, which provides more binding sites for metal ions, and the metal ions could accelerate the photoaging process [13]. Tang et al. observed increased levels of metal content, particularly iron and copper, which are believed to play a key role in hair photoaging through activating the production of hydroxyl radicals with dye [14]. Apart from hair dyeing, bleaching also leads to the increased copper ion adsorption of hair from tap water, which, in turn, results in the production of more oxidative free radicals. Naqvi et al. used probe technology to monitor hydroxyl radical formation in hair bleaching systems and found that the level of hydroxyl radicals formed under UV radiation was proportional to the level of copper present [15].

Aiming to protect hair from photoaging, different photoaging-preventing ingredients have been proposed by many researchers, which can be generally classified into three categories, including silicones, dyes, and antioxidants. Silicone, which is widely used in shampoo and other hair care products, could form a film on the hair surface, which can effectively prevent color change after UV radiation [16,17]. In addition to silicones, some dyes are also found to be able to protect hair against photoaging. Pande et al. found that the light absorption ability of the dyes can reduce the degradation of hair proteins under UV radiation, and some hair dyes can reduce the disulfide damage to 5% from 13% [18]. The utilization of antioxidants, which could quench free radicals or scavenge ROS (reactive oxygen species), is another approach to slowing down lipid oxidation and reducing protein degradation in photoaging. For example, Fernández et al. found that natural antioxidants obtained from artichoke and rice can prevent UV damage. Artichoke extract presented better performance in reducing lipid peroxidation and protein degradation, while rice extract was better at preserving the shine and color of dyed hair [19]. Similarly, pomegranate hydroalcoholic extract, honeysuckle extract, and tea extract have also been found to be effective in protecting the color of dyed hair and reducing protein loss under UV radiation, which may be related to their rich content of antioxidant active components such as tannins, flavonoids, and polyphenols [20,21].

Among diverse hair care ingredients, protein-based materials have been attracting more and more attention due to their excellent biocompatibility and ecological friendliness. Sahib et al. invented a peptide composition which can penetrate inside the human hair fiber to improve its strength and hydrophobicity [22]. Among protein-based materials, hydrolyzed keratin stands out as one of the most extensively studied components. This prominence is attributed not only to its readily available nature, but also to its chemical similarities with the keratin found in hair, which confers a higher compatibility and affinity with the hair’s own protein structure. Many studies have demonstrated that the inclusion of hydrolyzed keratin into hair care product formulations can markedly enhance the tensile strength of hair, efficiently prevent hair from breaking, and ameliorate the hygroscopic nature of hair, rendering hair more manageable [23,24]. However, the UV protection performance of hydrolyzed keratin has been rarely explored.

In this work, the performance of hydrolyzed keratin in protecting hair from photoaging was evaluated in terms of surface morphology and hair tensile test. The deposition and penetration behavior of hydrolyzed keratin were confirmed by fluorescence labeling experiments and Scanning Electron Microscopy (SEM) experiments. The roles of hydrolyzed keratin as a UV reducer and hair strengthener during UV radiation were demonstrated. Finally, based on the results of the stress relaxation test and Differential Scanning Calorimetry (DSC) analysis, a UV-induced degradation–penetration mechanism for the strengthening effect of hydrolyzed keratin on hair is proposed.

## 2. Result and Discussion

### 2.1. Deposition and Penetration Behavior of Hydrolyzed Keratin

**Figure 1:**
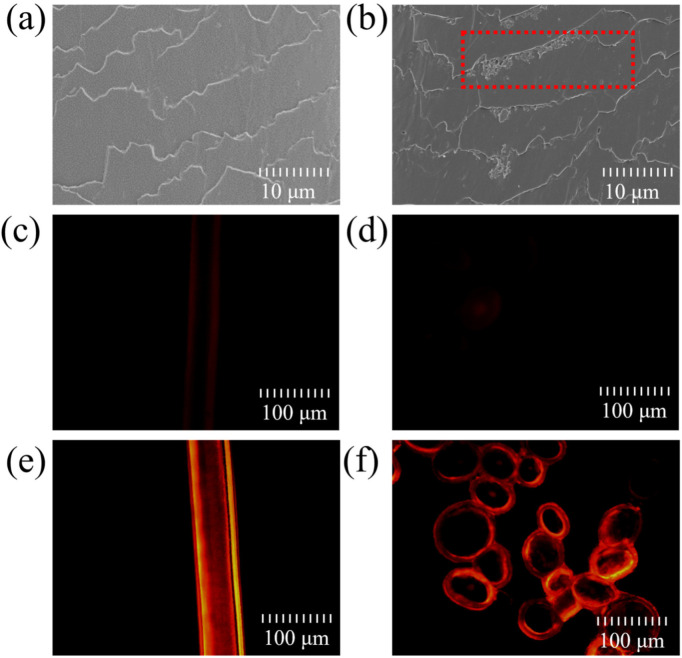
SEM pictures of hair cuticles (**a**) before and (**b**) after hydrolyzed keratin deposition, fluorescence pictures of hair (**c**) surface and (**d**) cross-section of control group, fluorescence pictures of hair (**e**) surface and (**f**) cross-section treated with labelled hydrolyzed keratin.

The deposition and penetration behavior of hydrolyzed keratin was investigated using SEM and a fluorescence microscope. The SEM images of untreated hair and hydrolyzed-keratin-treated hair samples are shown in Figure 1a,b, respectively. Comparing the two images, it can be observed that there was a significant deposition of keratin at the edges of the hair cuticle scales (the red box in Figure 1b), which is consistent with Malinauskyte’s findings [25]. Figure 1c,d shows the surface and cross-sectional images of hair samples treated with the free fluorescent dye after dialysis treatment, which presented a very subtle fluorescence. This distinctly differs from the orange-red fluorescence shown in Figure 1e,f, which verifies that hydrolyzed keratin can significantly deposit on the cuticle and penetrate into the hair cortex. Considering a moderate average molecular weight of about 3000 Da, the penetration and deposition of hydrolyzed keratin could both occur during the treatment. Some small molecules from the highly hydrolyzed keratin may permeate into the hair cortex, while the majority accumulate and deposit on the hair cuticle due to their large molecular weight. This finding is consistent with that of Malinauskyte in that mid-range MW hydrolyzed keratin could penetrate deeper into the cortex, and high-MW keratin peptides only penetrated the outer layers of the cortex [25].

The above results demonstrate that hydrolyzed keratin can effectively form a layer on the hair surface, which could serve as a UV reducer against environmental stressors, thereby enhancing the hair’s resilience and overall health. Similar conclusions are supported by many studies in the literature [26,27,28].

### 2.2. UV Protection Performance of Hydrolyzed Keratin

**Figure 2:**
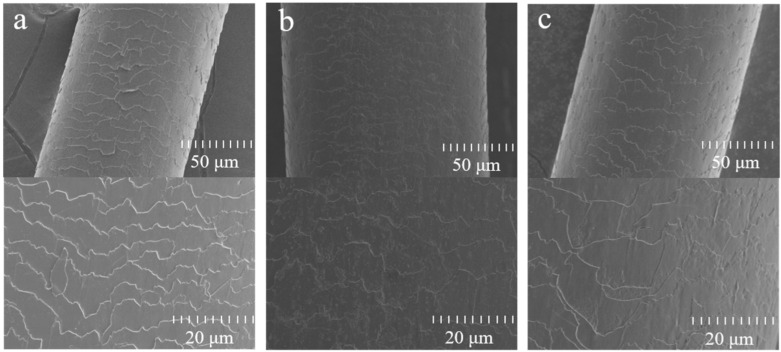
Morphology of cuticles characterized by SEM: (**a**) untreated hair before UV radiation, (**b**) untreated hair after UV radiation, and (**c**) hair treated with hydrolyzed keratin and then exposed to UV.

To investigate the photoprotective effect of hydrolyzed keratin on hair, hair samples treated with hydrolyzed keratin solution were bundled together and placed in a xenon lamp aging chamber for 6 days at 35 °C and 60% humidity, and the untreated hair served as the control group. As shown in Figure 2a,b, there was some cuticle erosion caused by UV radiation, making the hair cuticle rough and uneven. This finding is consistent with the literature [16,29]. However, as shown in Figure 2c, hair samples treated with hydrolyzed keratin only presented slight hair surface damage after exposure to UV radiation. The results prove that the hydrolyzed keratin film deposited on the hair surface can effectively prevent UV damage, playing the role as a UV reducer. (Additional images are available in Supplementary Materials (Figures S1–S3)).

**Figure 3:**
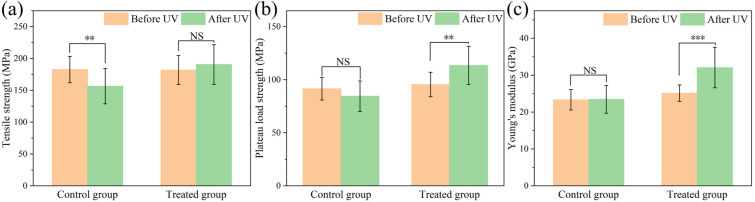
(**a**) Tensile strength, (**b**) plateau load, and (**c**) Young’s modulus of hair samples in the UV damage experiment. (NS: no significance; *p*: ** < 0.01, *** < 0.001).

The protective effect of hydrolyzed keratin on hair is also demonstrated by the outcomes of the tensile test. The tensile properties of hair samples were evaluated before and after UV radiation. As shown in Figure 3, photoaging lead to a significant decrease of 14.32% in tensile strength after UV radiation for hair without hydrolyzed keratin protection. As a UV reducer, hydrolyzed keratin helped prevent UV damage; hence, tensile strength did not decrease for the treated group after UV radiation. However, surprisingly, it was found that, after treatment with hydrolyzed keratin, hair samples exhibited a remarkable enhancement in plateau load strength and Young’s modulus properties after UV radiation, which presented an increase of 15.85% and 21.66%, respectively, after UV radiation. In comparison, before UV radiation, hair treated with hydrolyzed keratin presented similar tensile properties to the control group. The phenomena indicate that, besides being a UV reducer, the hydrolyzed keratin also plays a role as a hair strengthener, and this strengthening effect could protect the damaged hair from UV radiation.

### 2.3. Mechanism Investigation

**Figure 4:**
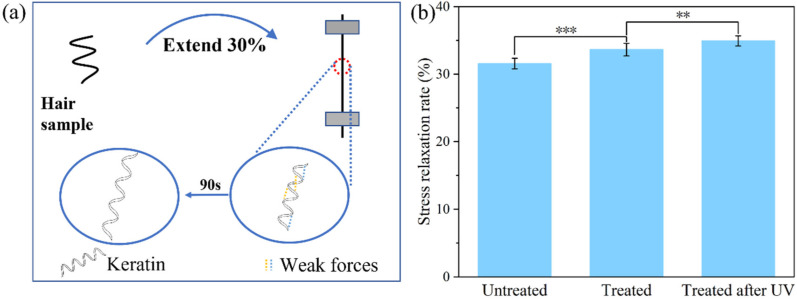
(**a**) Diagram of stress relaxation test, (**b**) proportion of weak interaction force (*p*: ** < 0.01, *** < 0.001).

To elucidate the mechanisms of the UV-induced strengthening effect of hydrolyzed keratin on hair, stress relaxation tests were conducted on hair samples before and after UV radiation. This test is usually employed to compare the proportion of weak intermolecular forces within different hair samples [30]. As illustrated in Figure 4a, during relaxation, weak chemical bonds including hydrogen and ionic bonds were broken within 90 s after the stress was applied, while strong chemical bonds such as covalent bonds remained. As depicted in Figure 4b, the treated group showed an enhancement in the proportion of weak chemical bonds by 6.56% compared with the untreated group, suggesting that the deposition and penetration of hydrolyzed keratin on hair enhances the internal chemical bonds within the hair. Moreover, after UV radiation, the treated group showed a further increase of 3.85% in the proportion of weak chemical bonds, indicating that UV radiation promoted the strengthening of the internal chemical bonds. Therefore, it can be concluded that hydrolyzed keratin not only acts as a UV reducer, which reduced hair damage, but also serves as a strengthener, enhancing the hair’s overall strength.

Therefore, when used as a treatment for hair, it acts as a strengthener, enhancing the hair’s overall strength. Furthermore, after UV radiation exposure, hydrolyzed keratin serves as a UV reducer, effectively reducing hair damage while also increasing the proportion of weak intermolecular forces in the hair, thus further enhancing its strength.

**Figure 5:**
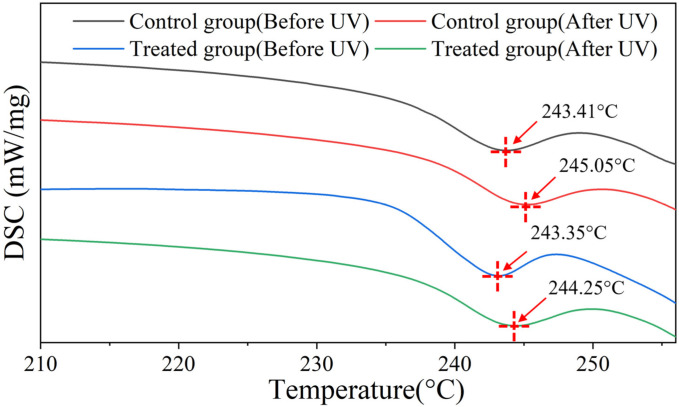
DSC curves of hair samples.

Thermal stability tests conducted on hair using DSC yielded comparable results, which are presented in Figure 5. It can be seen that, after UV radiation, the melting temperature of the control group shifted from 243.41 °C to 245.05 °C, which indicates more intermolecular forces created by fiber brittleness caused by UV [31]. In comparison, the group treated with hydrolyzed keratin protection also showed a smaller shift after UV radiation, with the peak position shifting from 243.35 °C to 244.25 °C. However, fiber brittleness seems unlikely for the treated group since the tensile properties did not show any reduction. Therefore, the peak shifting towards higher temperatures for the treated group should be ascribed to the formation of new intermolecular forces after UV irradiation, which is consist with the stress relaxation results.

**Figure 6:**
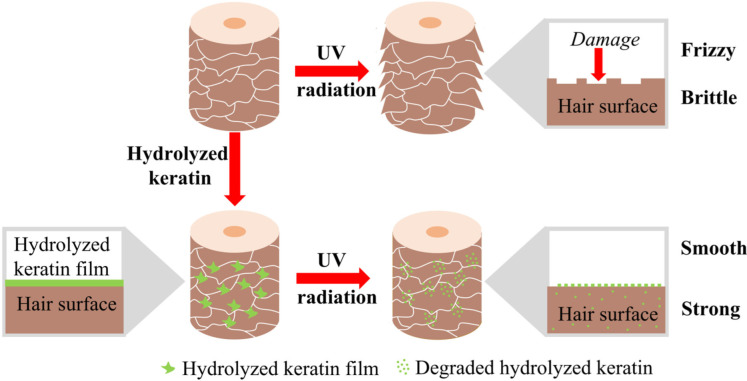
Mechanism diagram of hydrolyzed keratin’s protection on hair.

Combining the above experimental results, the UV protection provided by hydrolyzed keratin and its strengthening effect on hair after UV treatment are believed to mainly be contributed by the enhancement of intermolecular forces within hair fibers. This mechanism is similar to the repair mechanism of amino acids on damaged hair. Our previous study found that amino acids could improve the hair’s tensile strength and yield stress [32]. While considering the large molecular weight of hydrolyzed keratin, a UV-induced degradation–penetration mechanism is proposed. As illustrated in Figure 6, after treatment, hydrolyzed keratin can create a protective film on the hair surface. This film partly consumed UV and helped to maintain hair surface morphology under UV radiation. In addition, the film underwent degradation under UV radiation, which lead to a reduction in molecular weight and an increase in penetration ability. The degraded amino acids penetrated into the hair cortex and, therefore, enhanced the weak bonds within hair fibers, giving the hair surprisingly superior tensile properties compared to that prior to damage.

**Figure 7:**
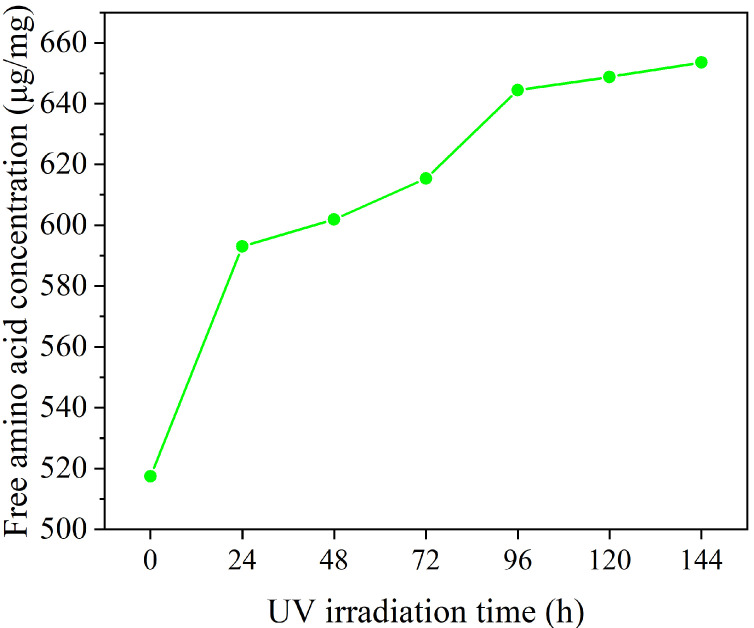
Free amino acid concentration after treatment with UV irradiation.

To verify this inference, hydrolyzed keratin solution was dried and subjected to UV radiation for a period of six days, during which the free amino acid content was monitored. As shown in Figure 7, the free amino acid concentration in the hydrolyzed keratin incrementally increased during UV irradiation, suggesting the UV-induced degradation of hydrolyzed keratin. The result suggests that large molecules in hydrolyzed keratin molecules were degraded into smaller peptides and amino acids under UV. As demonstrated by the experiments conducted by Malinauskyte [25], proteins of low molecular weight are more likely to penetrate into hair strands. Consequently, this UV-induced degradation process not only consumed the energy of UV, but also resulted in the formation of smaller molecular fragments that enhance penetration into the hair’s interior. This, in turn, contributes to a significant improvement in the hair’s tensile strength and overall resilience.

Although the UV-induced degradation–penetration mechanism is suggested by the above experiments, the direct observation of keratin degradation and amino acid formation on the hair surface is absent. Due to the composition similarity between hydrolyzed keratin and hair keratin, detecting the spectroscopic differences using microscopic infrared spectroscopy or Raman spectroscopy is quite challenging. Advanced technologies such as isotopic labeling and secondary ion mass spectrometry are promising methods that could further verify the degradation of hydrolyzed keratin and the penetration of the generated amino acids and provide in vivo experimental evidence [33,34,35,36].

## 3. Experimental Method

### 3.1. Materials

Sodium dodecyl sulfate (SDS), disodium hydrogen phosphate (Na_2_HPO_4_·12H_2_O), sodium dihydrogen phosphate (NaH_2_PO_4_·2H_2_O), and *N*,*N*-dimethylformamide(DMF): Sinopharm Chemical Reagent Co., Ltd. (Shanghai, China); 6-carboxytetramethylrhodamine and succinimidyl Ester (6-TAMRA, SE): Shanghai Yuanye Biotechnology Co., Ltd. (Shanghai, China); tissue freezing medium: Leica Biosystems (Nußloch, Germany); virgin white Chinese hair (18 cm × 1.5 cm × 1 g) was purchased from Shanghai Canyu Commercial Co., Ltd. (Shanghai, China), and sourced from Chinese females aged 20–60; hydrolyzed keratin (17.5%) and ProSina^TM^ were purchased from CRODA China (Shanghai, China); bleached hair samples (23 cm × 3 cm × 10 g) were sourced from Chinese females aged 20–60 and provided by Unilever (China) Investment Co., Ltd. (Shanghai, China).

Hydrolyzed-keratin-treated hair samples: bleached hair samples were soaked in the hydrolyzed keratin solution (2 wt%, with a pH of 5.5–6.0) for 4 h, and then placed at 25 °C and 60% RH for 24 h to remove the surface moisture.

Photoaging hair samples: hair samples were prepared into hair pieces and placed in a xenon lamp aging chamber to induce aging damage, simulating the natural damage that consumers’ hair experiences in daily life.

### 3.2. SEM Characterization

Surface morphology of hair samples was observed using a Field Emission Scanning Electron Microscope (S-4800, Hitachi, Tokyo, Japan) using a secondary electron mode at an accelerating voltage of 3 kV and a probe current of 10 μA. For each sample, at least 15 hair fibers were selected, trimmed, and attached to the sample stage using a conductive adhesive.

### 3.3. Hydrolyzed Keratin Permeability Test

The penetration behavior of hydrolyzed keratin was observed using a fluorescence labeling method, as detailed by Brunner [37]. Due to the fluorescence interference of melanin, white hair was employed in the test. Damaged white hair was obtained by bleaching the virgin Chinese white hair using powdered bleacher according to Imai [2]. Firstly, the bleached white hair was washed with 1% SDS solution and dried under constant temperature (25 °C ± 1 °C) and humidity (60% RH ± 5%). Then, the hair sample was soaked in a 2 wt% fluorescent-labeled hydrolyzed keratin solution for 4 h. The obtained hair sample was rinsed twice with deionized water and dried under constant temperature (25 °C ± 1 °C) and humidity (60% ± 5%). Next, the dried hair sample was completely covered with a tissue freezing agent and frozen overnight at −18 °C. The cryostat (CM1950, Leica, Nußloch, Germany) was used to cut the hair fibers into 25 μm sections. The fluorescence intensity was observed under the inverted biological microscope.

### 3.4. Tensile Tests

Tensile tests were conducted to investigate the restorative strength of hydrolyzed keratin solution on damaged hair using the fiber strength tester (XS (08) XT-3, Xusai, Shanghai, China). Before the test, hair samples were equilibrated at 25 °C and a humidity of 60% RH ± 5% for 24 h. The diameter of the hair was measured using a Laser Scan Micrometer (LSM-501S, Mitutoyo, Kawasaki, Japan). For each measurement, the hair fiber was rotated one full turn on the instrument to obtain the maximum and minimum readings, which were recorded as the major axis and minor axis of the hair section. The average diameter was then calculated by averaging the axis length values obtained from top, middle, and bottom of the hair. The tensile strength (*σ_t_*), plateau load ($\bar{\sigma }$), and Young’s modulus (*E*) of the single hair fiber were calculated using the following formulas [38,39]:

(1) $$\sigma_{t}=F_{b}/S$$

(2) $${\bar{\sigma }}_{t}=(F_{h}+F_{y})/(2\times S)$$

(3) $$E=(F_{h}/S)/(\Delta L/L)$$

where *F_b_*, *S*, *F_h_*, and *F_y_* are the breaking strength, cross-sectional area, yield strength, and yield zone endpoint strength of the fiber, and Δ*L* and *L* are the Hookean region length and initial length, respectively.

The results, based on 30 hair fibers for each group, were analyzed using a *t*-test to calculate the *p*-value with SPSS software(version 27.0, Chicago, IL, USA).

### 3.5. Stress Relaxation

The stress relaxation test was conducted on the same equipment as described in Section 3.4 using a similar method to that used by Barba et al [30]. Hair fibers with diameter of 70–90 μm were selected from hair samples. The selected single hair fiber was elongated at a constant ratio of 30% for 90 s and the stress was recorded as the relaxation curve. The stress curve (σ_r_), in the process, is calculated using the following formula [30]:

(4) $$\sigma_{r}=F/S$$

where *F* is the strength at 30% strain and *S* is the cross-section area of the hair. Then, the stress relaxation rate proportion *η* was calculated based on the stress value at 30% strain σ_0_ and the stress value σ_90_ at 90 s according to the following equation.

(5) $$\eta =(\sigma_{r0}-\sigma_{r90})/\sigma_{r0}$$

Results were based on 30 hair fibers, a *t*-test was performed for each group, and the *p* value was calculated using SPSS software.

### 3.6. DSC

DSC testing was conducted on a Differential Scanning Calorimeter (DSC 204 F1, NETZSCH, Selb, Germany). Before the test, hair samples were equilibrated at 25 °C and 60% RH for 24 h and then were cut into small pieces (about 5 mm); 10 mg was placed into an aluminum crucible, which was perforated for the easy removal of gases produced during heating. The temperature program was set to maintain a constant temperature at 60 °C for 10 min, followed by an increase to 260 °C at a rate of 10 °C/min, and nitrogen gas was used as the purge gas during the test [40].

### 3.7. Hydrolyzed Keratin Degradation Experiment

The hydrolyzed keratin solution was diluted to 2 wt% and equally divided into seven portions. All samples were dried in glass beakers using a vacuum drying oven (RSD-020Z, Royalstar, Hefei, China) to obtain dry films. The dry films were exposed under 365 nm UV radiation (PL-L 18W/10/4P, Philips, Amsterdam, The Netherlands) at 3.5 W/m^2^ for 6 days, simulating ten-day UV exposure (2 h/day) in Shanghai, China [41]. One sample was taken out every 24 h and subsequently dissolved in water and subjected to free amino acid testing using ninhydrin assay [42].

## 4. Conclusions

Upon the application of hydrolyzed keratin to hair, it forms a layer on the hair’s surface and partially permeates into the hair cortex. This film serves to mitigate the detrimental effects of UV radiation on hair, preserving the hair’s structural integrity and tensile and thermal stability. Additionally, it was found that hydrolyzed keratin treatment combined with subsequent UV radiation significantly improved the tensile properties of hair. The mechanism behind this involves the degradation of hydrolyzed keratin on the hair surface under UV radiation, resulting in the formation of smaller peptides or free amino acids. These lower-molecular-weight byproducts can penetrate the hair shaft, which, subsequently, leads to an enhancement in the hair’s tensile strength. This process underscores the role of hydrolyzed keratin in conferring resilience to the hair against environmental stressors, particularly those associated with UV exposure.

## Data Availability

Data are contained within the article and Supplementary Materials.

## Supplementary Materials

The following supporting information can be downloaded at: https://www.mdpi.com/article/10.3390/molecules30051182/s1, Figure S1: More pictures from same sample of Figure 2a. Figure S2: More pictures from same sample of Figure 2b; Figure S3: More pictures from same sample of Figure 2c.

## References

[1] Nogueira A.C.S., Nakano A.K., Joekes I. (2004). Impairment of hair mechanical properties by sun exposure and bleaching treatments. J. Cosmet. Sci..

[2] Imai T. (2011). The influence of hair bleach on the ultrastructure of human hair with special reference to hair damage. Okajimas Folia Anat. Jpn..

[3] Dyer J.M., Bell F., Koehn H., Vernon J.A., Cornellison C.D., Clerens S., Harland D.P. (2013). Redox proteomic evaluation of bleaching and alkali damage in human hair. Int. J. Cosmet. Sci..

[4] Contreras F., Ermolenkov A., Kurouski D. (2020). Infrared analysis of hair dyeing and bleaching history. Anal. Methods.

[5] Hyun J.W. (2023). Analysis of Morphological changes on the hair surface by heat perm treatment method. J. Korean Soc. Cosmetol..

[6] Slominski R.M., Chen J.Y., Raman C., Slominski A.T. (2024). Photo-neuro-immuno-endocrinology: How the ultraviolet radiation regulates the body, brain, and immune system. Proc. Natl. Acad. Sci. USA.

[7] Slominski A.T., Zmijewski M.A., Plonka P.M., Szaflarski J.P., Paus R. (2018). How UV light touches the brain and endocrine system through skin, and why. Endocrinology.

[8] Estibalitz F., Barba C., Alonso C., Martí M., Parra J.L., Coderch L. (2012). Photodamage determination of human hair. J. Photochem. Photobiol. B Biol..

[9] Robbins C.R., Bahl M.K. (1984). Analysis of hair by electron spectroscopy for chemical analysis. J. Cosmet. Sci..

[10] Ji J.H., Park T.S., Lee H.J., Kim Y.-D., Pi L.-Q., Jin X.-H., Lee W.-S. (2013). The ethnic differences of the damage of hair and integral hair lipid after ultra violet radiation. Ann. Dermatol..

[11] Hoting E., Zimmermann M., Höcker H. (1995). Photochemical alterations in human hair. II: Analysis of melanin. J. Cosmet. Sci..

[12] Richena M., Rezende C.A. (2015). Effect of photodamage on the outermost cuticle layer of human hair. J. Photochem. Photobiol. B Biol..

[13] Smart K.E., Kilburn M., Schroeder M., Martin B.G.H., Hawes C., Marsh J.M., Grovenor C.R.M. (2009). Copper and calcium uptake in colored hair. Int. J. Cosmet. Sci..

[14] Tang Y., Dyer J.M., Deb-Choudhury S., Li Q. (2016). Trace metal ions in hair from frequent hair dyers in China and the associated effects on photo-oxidative damage. J. Photochem. Photobiol. B Biol..

[15] Naqvi K.R., Marsh J.M., Godfrey S., Davis M.G., Flagler M.J., Hao J., Chechik V. (2013). The role of chelants in controlling Cu (II)-induced radical chemistry in oxidative hair colouring products. Int. J. Cosmet. Sci..

[16] Michelli F.D., André R.B., Maria V.R.V. (2015). Effects of solar radiation on hair and photoprotection. J. Photochem. Photobiol. B Biol..

[17] Schlosser A. (2004). Silicones used in permanent and semi-permanent hair dyes to reduce the fading and color change process of dyed hair occurred by wash-out or UV radiation. J. Cosmet. Sci..

[18] Pande C.M., Albrecht L., Yang B. (2001). Hair photoprotection by dyes. J. Cosmet. Sci..

[19] Fernández E., Martínez-Teipel B., Armengol R., Barba C., Coderch L. (2012). Efficacy of antioxidants in human hair. J. Photochem. Photobiol. B Biol..

[20] Dario M.F., Pahl R., de Castro J.R., de Lima F.S., Kaneko T.M., Pinto C.A., Baby A.R., Velasco M.V.R. (2013). Efficacy of *Punica granatum L.* hydroalcoholic extract on properties of dyed hair exposed to UVA radiation. J. Photochem. Photobiol. B Biol..

[21] Davis S.L., Marsh J.M., Kelly C.P., Li L., Tansky C.S., Fang R., Simmonds M.S.J. (2022). Protection of hair from damage induced by ultraviolet irradiation using tea (*Camellia sinensis*) extracts. J. Cosmet. Dermatol..

[22] Sahib S., Jungman E., Aquis Hairsciences Inc (2020). Composition for Improving Hair Health.

[23] Cruz C.F., Azoia N.G., Matamá T., Cavaco-Paulo A. (2017). Peptide-protein interactions within human hair keratins. Int. J. Biol. Macromol..

[24] Tinoco A., Gonçalves J., Silva C., Loureiro A., Gomes A.C., Cavaco-Paulo A., Ribeiro A. (2018). Keratin-based particles for protection and restoration of hair properties. Int. J. Cosmet. Sci..

[25] Malinauskyte E., Shrestha R., Cornwell P.A., Gourion-Arsiquaud S., Hindley M. (2021). Penetration of different molecular weight hydrolysed keratins into hair fibres and their effects on the physical properties of textured hair. Int. J. Cosmet. Sci..

[26] Wiesche E.S., Körner A., Schäfer K., Wortmann F.J. (2011). Prevention of hair surface aging. J. Cosmet. Sci..

[27] Camargo F.B., Minami M.M., Rossan M.R., Magalhães W.V., Porto Ferreira V.T., Maia Campos P.M.B.G. (2022). Prevention of chemically induced hair damage by means of treatment based on proteins and polysaccharides. J. Cosmet. Dermatol..

[28] Cavallaro G., Milioto S., Konnova S., Fakhrullina G., Akhatova F., Lazzara G., Fakhrullin R., Lvov Y. (2020). Halloysite/keratin nanocomposite for human hair photoprotection coating. ACS Appl. Mater. Interfaces.

[29] Maeda K., Yamazaki J., Okita N., Shimotori M., Igarashi K., Sano T. (2018). Mechanism of cuticle hole development in human hair due to UV-radiation exposure. Cosmetics.

[30] Barba C., Scott S., Roddick-Lanzilotta A., Kelly R., Manich A.M., Parra J.L., Coderch L. (2010). Restoring important hair properties with wool keratin proteins and peptides. Fibers Polym..

[31] Chandrashekara M.N., Ranganathaiah C. (2010). Chemical and photochemical degradation of human hair: A free-volume microprobe study. J. Photoch. Photobio. B.

[32] Fan J., Yu W., Bian M., Yue Z., Wang J., Chang K. (2024). Study on the efficacy and mechanism of an amino acid combination in hair care. China Surfactant Deterg. Cosmet..

[33] Masayuki O., Kazutaka I., Noriyuki T., Aoyagi S. (2012). Investigation of the damage on the outermost hair surface using ToF-SIMS and XPS. Surf. Interface Anal..

[34] Flinders B., Cuypers E., Zeijlemaker H., Tytgat J., Heeren R.M.A. (2015). Preparation of longitudinal sections of hair samples for the analysis of cocaine by MALDI-MS/MS and TOF-SIMS imaging. Drug Test. Anal..

[35] Kempson I.M., Skinner W.M. (2005). ToF-SIMS analysis of elemental distributions in human hair. Sci. Total Environ..

[36] Henderson G.L., Harkey M.R., Chihong Z., Jones R.T., Jacob P. (1996). Incorporation of isotopically labeled cocaine and metabolites into human hair: 1. dose-response relationships. J. Anal. Toxicol..

[37] Brunner A., Minamitake Y., Göpferich A. (1998). Labelling peptides with fluorescent probes for incorporation into degradable polymers. Eur. J. Pharm. Biopharm..

[38] Antunes E., Cruz C.F., Azoia N.G., Cavaco-Paulo A. (2016). Insights on the mechanical behavior of keratin fibrils. Int. J. Biol. Macromol..

[39] Wortmann F.J., Quadflieg J.M., Wortmann G. (2022). Comparing hair tensile testing in the wet and the dry state: Possibilities and limitations for detecting changes of hair properties due to chemical and physical treatments. Int. J. Cosmet. Sci..

[40] Lima C.R.R.d.C., Machado L.D.B., Velasco M.V.R., Matos J.D.R. (2018). DSC measurements applied to hair studies. J. Therm. Anal. Calorim..

[41] Gu H.j., Peng L., Jiang W.C., Tan Y.-M., Zhou G.-J., Kan H.-D., Chen R.-J., Zou Y. (2021). Impact of solar ultraviolet radiation on daily outpatient visits of atopic dermatitis in Shanghai, China. Environ. Sci. Pollut. Res..

[42] Abernathy D.G., Spedding G., Starcher B. (2009). Analysis of protein and total usable nitrogen in beer and wine using a microwell ninhydrin assay. J. Inst. Brew..

