# Supplementary material for: Performance and Mechanism of Hydrolyzed Keratin for Hair Photoaging Prevention
Source: Molecules. 2025 Mar 6;30(5):1182. doi: 10.3390/molecules30051182 (PMC11902160; doi:10.3390/molecules30051182)
Supplement: Supplementary file 1 [file molecules-30-01182-s001.zip › molecules-3428313-supplementary.pdf]

molecules

Supplementary Material

Performance and mechanism of hydrolyzed keratin for hair photoaging prevention

Jiayi Fan†[1], Lei Wu†[1], Jing Wang,[1] Xiaoying Bian[2], Chongchong Chen[2], Kuan Chang[1,*]

[1] Key Laboratory of Synthetic and Biological Colloids, Ministry of Education, School of Chemical & Material Engineering, Jiangnan University, Wuxi, Jiangsu, 214122, China;

[2] Unilever (China) Investing Co. Ltd., Shanghai, 200335, China

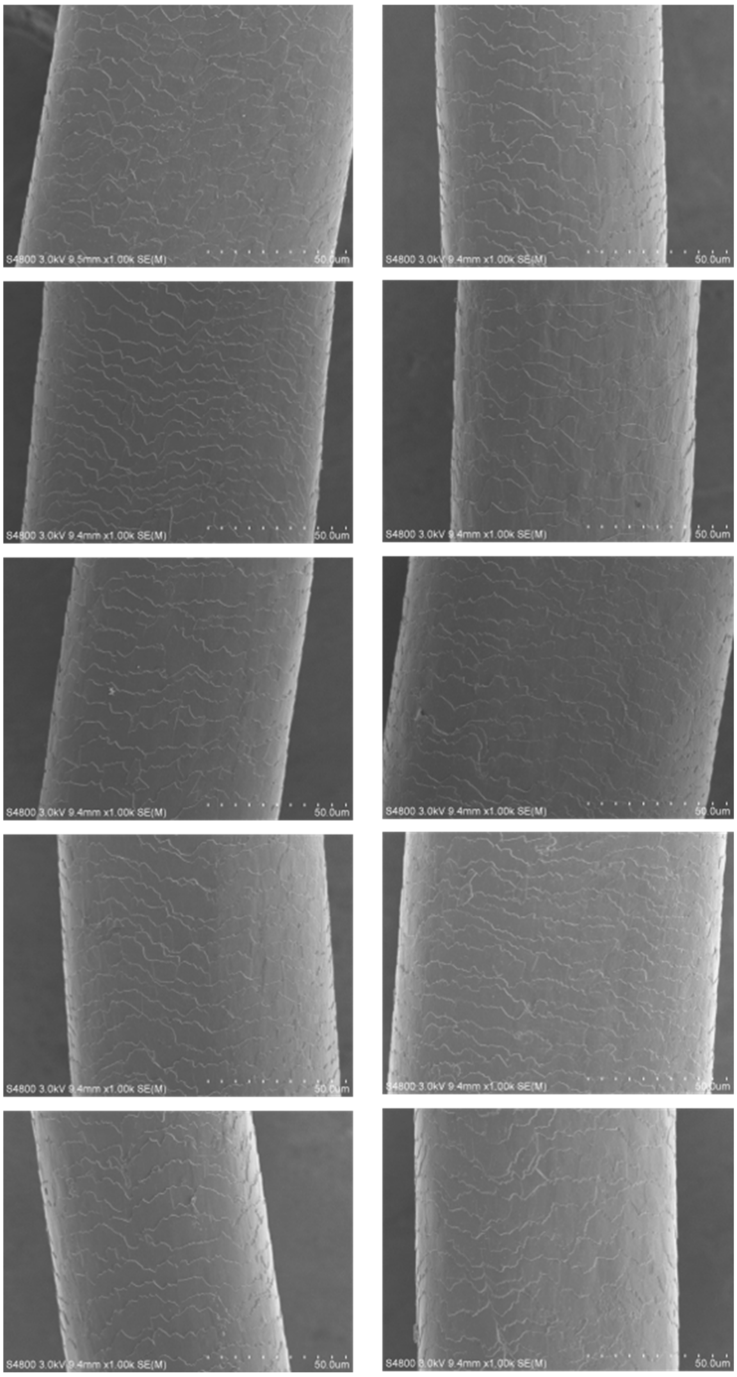

Figure. S1 more pictures from same sample of Figure.2(a)

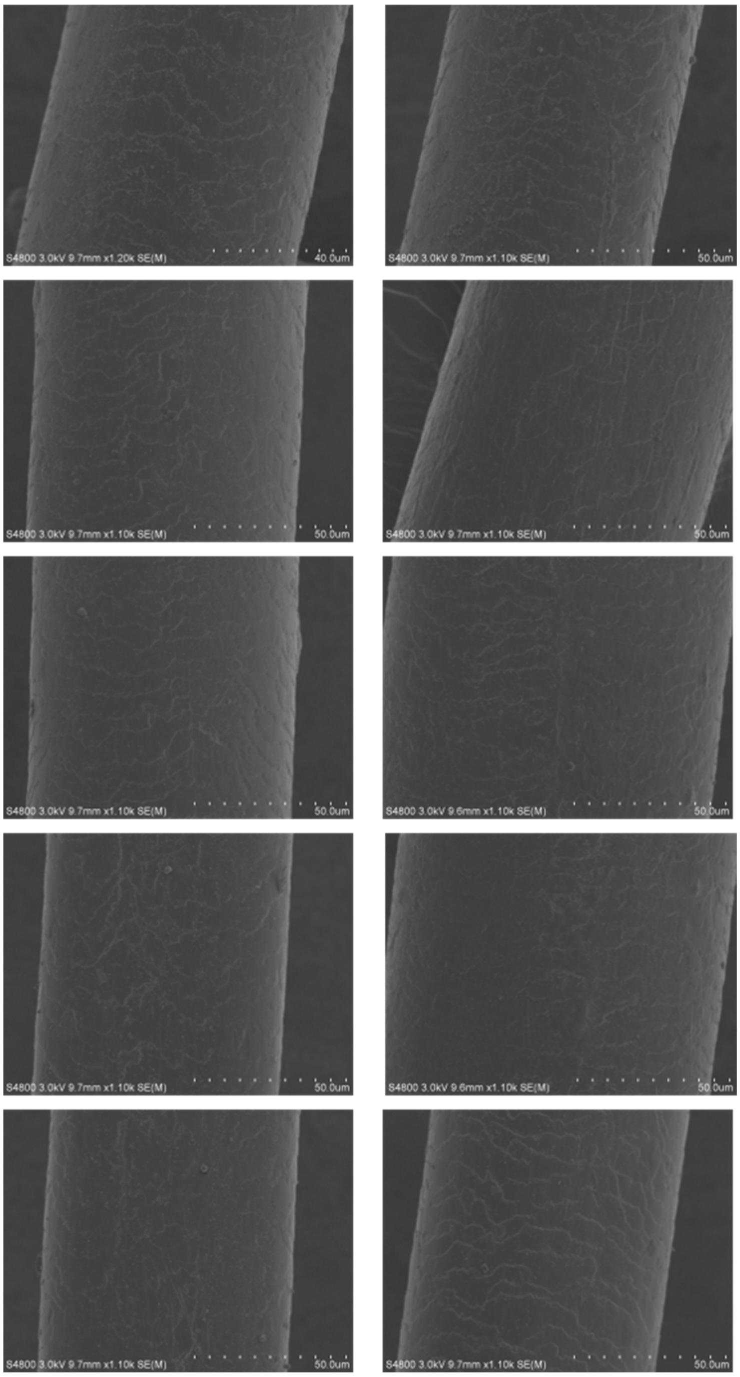

Figure.S2 more pictures from same sample of Figure.2(b)

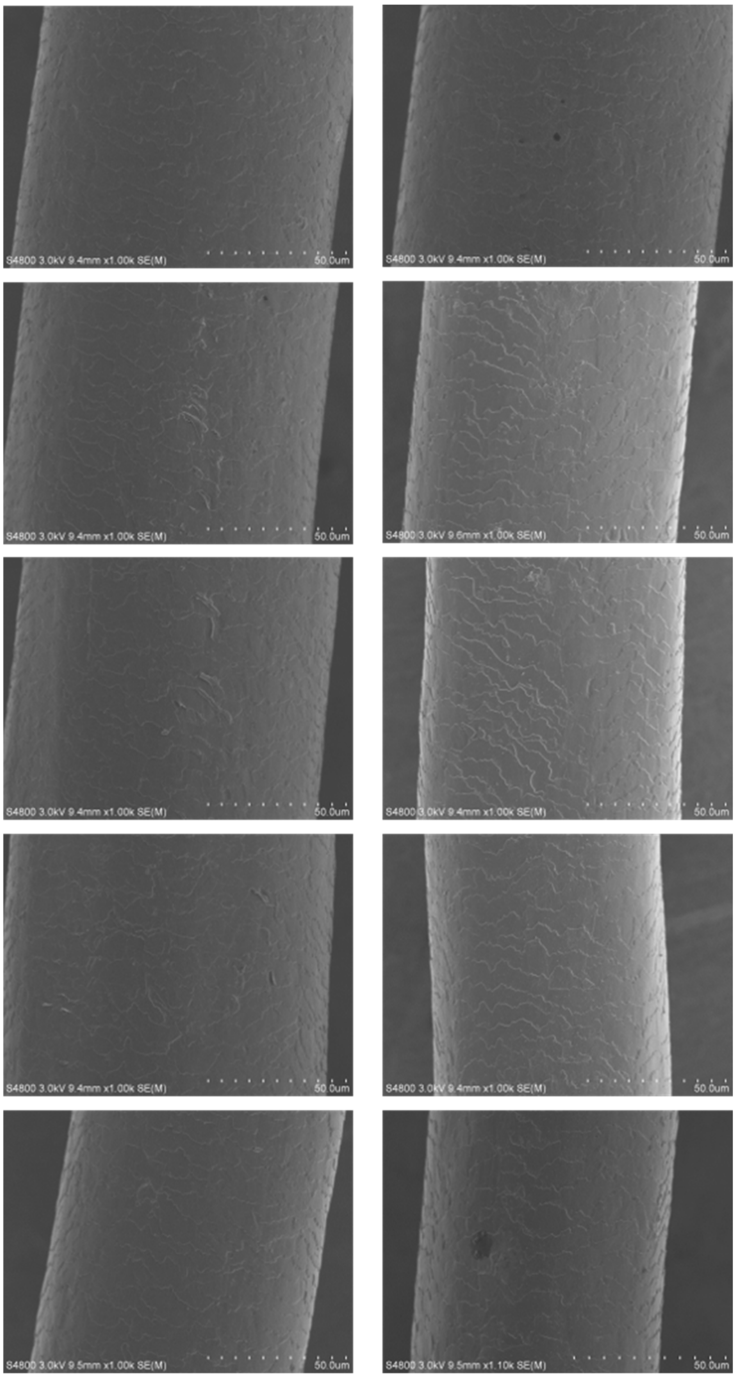

Figure.S3 more pictures from same sample of Figure.2(c)
